# Inpatients with severe-enduring anorexia nervosa: Understanding the “enduringness” specifier

**DOI:** 10.1192/j.eurpsy.2021.2218

**Published:** 2021-06-21

**Authors:** Enrica Marzola, Matteo Martini, Annalisa Brustolin, Giovanni Abbate-Daga

**Affiliations:** Eating Disorders Center, Department of Neuroscience “Rita Levi Montalcini”, University of Turin, Turin, Italy

**Keywords:** eating disorders, hospitalization, quality of life, severe-enduring, stages of change

## Abstract

**Background:**

Despite the need for a common definition of severe and enduring anorexia nervosa (SE-AN) with the overarching goal to optimize treatments, this definition still is being debated. Therefore, in this study we conducted an in-depth investigation of the history of AN and its clinical outcomes on inpatients with AN to ascertain the eventual “profiles” for individuals with varying durations of the illness (DOI).

**Methods:**

We recruited 169 inpatients with AN, grouping them according to DOI: <3 years (short duration, SD-AN); 3–6.99 years (medium duration, MD-AN); and ≥7 years (long duration, LD-AN). We then performed a self-report and interview-based investigation of AN history, clinical data, eating, and general psychopathology, including personality, premorbid traits, stage of change, and quality of life. We measured the clinical outcomes for hospitalization as well.

**Results:**

The majority of the measures did not differ across groups. Those with LD-AN were older and diagnosed mostly with the binge-purging AN subtype, failed more previous AN-related treatments, reported a lower lifetime body mass index, and trended toward a younger age at onset when compared to the other groups. All patients responded equally well to hospitalization, but patients with SD-AN improved less in drive for thinness and body-related concerns.

**Conclusions:**

We did not find the “enduringness” of AN to be a specifier of severity. Hospitalization was effective for those with LD-AN and MD-AN, while interventions for the core cognitive aspects of over-evaluation of body shape should be offered to patients with SD-AN.

## Introduction

Only 60% of patients with anorexia nervosa (AN) fully recover over the long-run [1] while a substantial number of patients develop a severe and enduring course (SE-AN). If this is the case, patient clinical history is characterized by relevant medical and psychological comorbidities, poor quality of life (QOL), and treatment resistance [1,2]. Importantly, a prolonged duration of illness (DOI) entails interpersonal and behavioral consequences coupled with chronic stress, thus constituting a maintaining factor [3].

Recent research has authoritatively highlighted the need for “data to clarify the nature and boundaries of SE-AN” [4]. The definitions and cutoffs for AN are challenging [5] and studies have proposed that DOI cannot be a specifier of severity because some patients need many years to recover [6,7]. Only a few studies have tried to classify SE-AN by comparing patients with different DOI or treatment responses. Data from Wildes et al. [8] did not support patients’ DOI and body mass index (BMI) as “defining” SE-AN, suggesting instead a role for eating disorder behaviors and QOL. More recently, Ambwani et al. [9] have found that patients with AN with different DOI had no differences in BMI or eating psychopathology after receiving outpatient treatment, but those with a longer DOI reported lower improvement in work or social adjustment.

However, the issue is more complicated when patients with SE-AN need to be hospitalized because of acute illness. In fact, those requiring hospitalization usually battle myriad medical, relational, and psychiatric needs [4], leading to a five times increased mortality risk [10]. Therefore, other severity indexes have been proposed, including QOL, motivation to recover, and number of previous treatment attempts [11]. Several different DOI cutoffs have been proposed for defining SE-AN, including 3 [5], 5 [12], 7 [7,9,13], 10 [14], and more than 20 years [15] with a recent review finding a mode of 7 years [11].

Given the inadequacy of DOI to describe patients with SE-AN [8], it has been suggested that a putative definition should also include treatment resistance [5]. Similarly, a detailed description of treatment mechanisms coupled with a broad reconceptualization of outcomes has been advocated for eating disorders [16]. Consequently, the most recent definition of SE-AN reads as a persistent state of dietary restriction, being underweight, and overvaluation of weight and shape with functional impairment, coupled with at least 3 years of AN and exposure to at least two evidence-based treatments [5].

However, the relationship between SE-AN and treatment feasibility is complex and plagued by several issues. First, factual treatment options for patients with SE-AN range from medical palliative care [17] to neuromodulation [18], with an open debate on the most appropriate focus for psychological therapies [4]. Second, the failure of previous therapeutic attempts is a vague concept, with “nonresponse” (e.g., relapse after treatment) confused with “treatment resistance” (e.g., not improving after a certain therapy or refusing treatments). Third, certain labels can affect treatment availability for patients with SE-AN. An enduring course of AN could entail different (and sometimes poorer [19]) possibilities of access to care, according to each health care system. Therefore, a common definition of SE-AN is needed not only for patients and their families but also for clinicians and researchers.

Despite previous research efforts, data are still conflicting and largely lacking about inpatients with SE-AN. Therefore, we aimed to investigate both the history of AN (and AN-related treatments) and the outcomes of hospitalization using a large sample of inpatients with dissimilar DOI. We had a twofold aim: first, to precisely measure the “profile” of SE-AN by performing a comprehensive assessment of patients with different DOI (e.g., history of AN, clinical data, eating and general psychopathology, personality, premorbid traits, stage of change, and QOL). Second, we aimed to shed light on the potentially diverse clinical trajectories of hospitalization of patients with different DOI. As inpatients with AN might entail a different severity scenario than that mentioned in the findings reported so far, for those with a longer DOI, we expected to find a greater overall severity coupled with poorer outcomes on hospital discharge.

## Methods

### Participants

A total of 190 inpatients were consecutively enrolled at the Psychiatry Ward of the Eating Disorders Center of the “Città Della Salute e Della Scienza” Hospital of the University of Turin, Italy, from January 2016 to June 2020. All participants were Caucasian and voluntarily admitted. Inclusion criteria were: (a) diagnosis of AN as assessed on admission by an experienced psychiatrist using a structured interview [20]; (b) age range of 16–55 years; and (c) no history of psychotic-spectrum disorders, bipolar disorders, or substance use dependence. In our analyses, we used a patient’s first admission to our unit in cases of multiple hospitalizations.

Of the initial sample, five patients refused to provide their consent to participate in the study, and 16 returned incomplete assessments; therefore, the final sample was 169 inpatients. All patients provided written informed consent for this Ethical Committee-approved study (Approval No. 0036472).

To ensure data comparability, using the earlier literature [9,11] we divided the sample as follows: patients with short (SD-AN, <3 years), medium (MD-AN, 3–6.99 years), and long DOI (LD-AN, ≥7 years). During the admission interview, an experienced psychiatrist measured patients’ DOI, documenting the first onset of the eating disorder. No patients reported lifetime cross-over across diagnoses of eating disorders, so the data collected for our sample consistently referred to the DOI of AN.

### Procedures

We collected the patients’ clinical characteristics at hospital admission (T0) and discharge (e.g., end of treatment [EOT]). An experienced psychiatrist performed a specific anamnestic investigation on patient DOI and previous treatments to disentangle nonresponse versus treatment resistance. We calculated BMI at both time points.

All patients completed the following assessments at T0 and EOT: the *Eating Disorder Inventory-2 (EDI-2)*, Italian version [21], to measure eating disorder attitudes and behaviors (we included in our analysis the “core” subscales of this questionnaire, namely drive for thinness, bulimia, and body dissatisfaction); the *Eating Disorder Examination Questionnaire (EDE-Q)*, Italian version [22], to assess eating psychopathology; the *Body Shape Questionnaire (BSQ)* [23], to estimate body image perception; the *State–Trait Anxiety Inventory (STAI)*, Italian version [24], to assess state and trait anxiety; the *Beck Depression Inventory (BDI)* [25] to measure depressive symptoms; the *Hamilton Rating Scale for Anxiety (HAM-A)* [26] to assess severity of anxiety through symptom gradation; the *Hamilton Depression Rating Scale (HAM-D)* [27] to investigate depressive symptoms; and the *EuroQoL Health Questionnaire/Visual Analogue Scale (EQ-5D)* [28] to measure QOL.

All patients completed the following assessments at T0: the *Anorexia Nervosa Stages of Change Questionnaire (ANSOCQ)* [29] to evaluate the core features of AN and assess stages of change; the *Temperament Evaluation of Memphis, Pisa, Paris and San Diego Autoquestionnaire (TEMPS-A)*, Italian version [30], to investigate affective temperaments; the *Frost Multidimensional Perfectionism Scale (FMPS)* [31] to evaluate multidimensional perfectionism; the *Childhood Retrospective Perfectionism Questionnaire (CHIRP)-self-report version* [32] to assess childhood AN-related traits; and the *Premorbid Childhood Traits Questionnaire (PCT-Q)* [33] to measure childhood harm avoidance, reward sensitivity, social phobia, alexithymia, achievement drive, interoceptive awareness, food obsessions, worry about future, and sleep problems.

At EOT all patients completed the *Working Alliance Inventory-Short Revised (WAI-SR)* [34] to evaluate therapeutic alliance.

### Treatment

All participants were inpatients seeking treatment at the Eating Disorders Center of the University of Turin, Italy, with the vast majority admitted through the emergency room in an acute phase of AN. All treatment costs were covered by the National Health System, so no financial or insurance barriers existed for the patients. The treatment was delivered following international guidelines [35]. The intervention was “noneclectic”; it was integrated and focused on improving patients’ AN-related life-threatening conditions and managing eating symptoms. The intervention had the following aims: (a) to reduce patients’ clinical life-threatening conditions; (b) to foster patients’ motivation for the subsequent therapeutic steps; (c) to work with structured daily sessions on symptom management focusing on diet and body image; (d) to work psychologically to understand the possible causes that led to an emergency admission; and (e) to provide families with psychoeducation. We strictly monitored patients from a medical standpoint and provided psychological interventions informed by motivational interviewing to support patients’ daily efforts and to reinforce the next steps of treatment. No involuntary admissions are allowed at our unit nor are coercive methods used. Instead, we offer behavioral contracting for meals and eating symptoms.

### Statistical analysis

We used the SPSS 26.0 statistical software package (IBM SPSS Statistics for Windows, version 26.0; IBM Corp., Armonk, NY) for data analysis. We split the sample into three groups according to the aforementioned criteria (SD-AN, <3 years; MD-AN, 3–6.99 years; LD-AN, ≥7 years). Subsequently, we conducted an analysis of variance (ANOVA), followed by the Sheffè post hoc test, to ascertain differences across groups in the continuous variables. For categorical parameters we used Fisher’s exact test with correction for multiple comparisons. To investigate hospitalization outcomes and differences across groups over time, we performed a repeated-measures ANOVA. We measured effect size using eta-squared (*η* ^2^) according to Cohen’s work [36] on effect sizes: small, *η* ^2^ = 0.01–0.06; moderate, *η* ^2^ = 0.06–0.14; or large, *η* ^2^ > 0.14.

## Results

### Sociodemographic and clinical characteristics

Patients with LD more frequently were married and unemployed than were those with SD and MD (Table 1). Patients with MD and LD were more often diagnosed with AN-BP than were patients with SD but also reported more diagnostic cross-over. Age significantly differed across groups, with patients with SD being younger than those with MD, who in turn were younger than those LD. Also, the SD group reported a lowest lifetime BMI significantly higher than the LD group; however, comparisons between the SD and MD groups and the MD and LD groups did not differ from each other. Similarly, patients with SD reported a significantly lower number of AN-related hospitalizations than did those with LD; however, comparisons between patients with SD and MD and with MD and LD did not differ from each other. Those with LD also showed a trend toward a significantly earlier age at onset when compared to those with SD. In contrast, the groups did not differ in gender distribution, current BMI, medications at admission, family history of psychiatric disorders (including AN), and past sexual abuse and suicide attempts.

**Table 1.** Differences in sociodemographic and clinical data for patients with short, medium, and long duration of illness.

	Total sample inpatients with AN *n* = 169								
	<3 years		≥3 and <7 years		≥7 years		Test statistics		
	Short duration (SD-AN) *n* = 76		Medium duration (MD-AN) *n* = 47		Long duration (LD-AN) *n* = 46				
	Mean (SD)	*N* (%)	Mean (SD)	*N* (%)	Mean (SD)	*N* (%)	*F*	*p*	Sheffè post hoc
Age, years	19.6 (4.9)		23.1 (7.5)		31.7 (9.4)		41.75	**<0.001**	SD-AN < MD-AN < LD-AN
Gender								0.735^a^	
Female		71 (93.4)		43 (91.5)		44 (95.7)			
Male		5 (6.6)		4 (8.5)		2 (4.3)			
Status								**0.001** ^a^	
Single		66 (86.8)^b^		40 (85.1)^b^		27 (58.7)^c^			
In a relationship/married		10 (13.2)^b^		7 (14.9)^b^		17 (37)^c^			
Divorced		0 (0)^b^		0 (0)^b^		2 (4.3)^b^			
Employment								**<0.001** ^a^	
Student		34 (44.7)^b^		19 (40.4)^b^		5 (10.9)^b^			
Employed		33 (43.4)^b^		18 (38.3)^b^		23 (50)^b^			
Unemployed		9 (11.9)^b^		10 (21.3)^b,c^		18 (39.1)^c^			
Subtype of AN								**0.006** ^a^	
AN-R		65 (85.5)^b^		31 (66)^c^		29 (63)^c^			
AN-BP		11 (14.5)^b^		16 (34)^c^		17 (37)^c^			
Diagnostic cross-over in the past		3 (4)^b^		7 (14.9)^b,c^		9 (20)^c^		**0.014** ^a^	
Age at onset, years	18.3 (5.2)		18.9 (7.5)		15.8 (4.1)		3.159	**0.046**	SD-AN = MD-AN
									MD-AN = LD-AN
									SD-AN > LD-AN^d^
Body mass index	14.4 (1.9)		14.4 (1.7)		14.3 (2.1)		0.007	0.993	–
Lowest lifetime BMI	14.1 (2)		13.7 (1.6)		12.8 (1.9)		5.91	**0.003**	SD-AN = MD-AN
									MD-AN = LD-AN
									SD-AN > LD-AN
Previous AN-related hospitalizations		21 (31.3)^b^		23 (48.9)^c^		29 (63)^c^		**<0.001** ^a^	
Number of previous hospitalizations	0.4 (.8)		1.1 (1.6)		2.6 (3.2)		17.91	**<0.001**	SD-AN = MD-AN
									MD-AN = LD-AN
									SD-AN < LD-AN
Use of medications upon admission		44 (57.9)		31 (66)		30 (65.2)		0.585^a^	
Ineffective therapeutic strategies before admission									
Medications		14 (18.4)^b^		14 (30.4)^c^		25 (54.3)^c^		**0.001** ^a^	
Psychotherapy		24 (31.6)^b^		27 (57.4)^c^		29 (63)^c^		**0.002** ^a^	
History of sexual abuse		4 (5.3)		6 (12.8)		6 (13)		0.212^a^	
Family history of psychiatric disorders		22 (28.9)		14 (30.4)		17 (37)		0.688^a^	
Family history of eating disorders		11 (14.5)		9 (19.1)		8 (17.4)		0.805^a^	
History of previous suicide attempts		13 (17.1)		13 (27.7)		10 (21.7)		0.359^a^	

a *p*-value of the Fisher’s exact test.

d *p*-value = 0.066.

The sample included 33.7% diagnosed with major depression, with no differences across groups. Anxiety disorders were present in 9.5% of the sample, with those with LD showing significantly more anxious comorbidity than did those with SD (SD, 5.3%; MD, 6.4%; LD, 19.6%; *p* = 0.030).

### Eating and general psychopathology, stages of change, and QOL

For eating and general psychopathology, the three groups differed only on the EDI-2 bulimia subscale, with the SD group reporting the lowest scores. No differences emerged for anxiety and depression, self-esteem, AN stages of change, and QOL (Table 2).

**Table 2.** Eating and general psychopathology, stages of change, and quality of life across groups of patients with anorexia nervosa (AN) showing short, medium, or long duration of illness.

	Total sample inpatients with AN *n* = 169					
	<3 years	≥3 and <7 years	≥7 years	Test statistics		
	Short duration (SD-AN) *n* = 76	Medium duration (MD-AN) *n* = 47	Long duration (LD-AN) *n* = 46			
	Mean (SD)	Mean (SD)	Mean (SD)	*F*	*p*	Sheffè post hoc
EDI-2						
Drive for thinness	12.8 (7.9)	10.8 (7.9)	11.4 (7.9)	0.86	0.426	–
Bulimia	2.2 (3.5)	3.3 (4.9)	5.1 (6.6)	4.2	**0.017**	SD-AN = MD-AN
						MD-AN = LD-AN
						SD-AN < LD-AN
Body dissatisfaction	13.7 (6.7)	13.4 (6.9)	14.5 (7)	0.29	0.75	–
EDE-Q						
Restraint	3.3 (1.9)	2.7 (2)	3.1 (2.2)	0.91	0.407	–
Eating concern	3.1 (1.5)	2.7 (1.6)	2.7 (1.9)	0.81	0.447	–
Shape concern	3.9 (1.7)	4.1 (1.7)	3.7 (1.8)	0.54	0.585	–
Weight concern	3.5 (1.8)	5.6 (1.6)	3.2 (1.9)	0.37	0.694	–
Total	3.4 (1.6)	3.2 (1.6)	3.2 (1.8)	0.38	0.68	–
BSQ	102 (43.7)	113.6 (42.7)	113.3 (54.1)	0.35	0.702	–
STAI						
State	54.3 (14.8)	53.7 (13.9)	54.4 (13.7)	0.03	0.968	–
Trait	55.9 (15.3)	58.6 (11.4)	59.7 (11.9)	1.1	0.338	–
HAM-A	19.9 (7.2)	17.8 (6.6)	18.8 (6.4)	1.198	0.305	
BDI	16.2 (8.4)	16.8 (7.3)	15.2 (8.1)	0.39	0.681	–
HAM-D	20.3 (7.4)	18.5 (7.5)	17.7 (7.5)	1.718	0.183	
ANSOCQ	2.6 (0.9)	2.7 (0.7)	2.6 (0.8)	0.24	0.788	–
EQ-5D						
Index	0.4 (0.5)	0.5 (0.4)	0.4 (0.6)	0.46	0.630	–
EQ5D-VAS	53.9 (24.7)	46.7 (20.1)	44.8 (24.6)	2.1	0.132	–

Abbreviations: ANSOCQ, Anorexia Nervosa Stages of Change Questionnaire; BDI, Beck Depression Inventory; BSQ, Body Shape Questionnaire; EDE-Q, Eating Disorders Examination-Questionnaire; EDI-2, Eating Disorder Inventory-2; EQ-5D, EuroQoL Health Questionnaire/Visual Analogue Scale; HAM-A, Hamilton Anxiety Rating Scale; HAM-D, Hamilton Depression Rating Scale; STAI, State Trait Anxiety Inventory.

### Personality and premorbid traits

All patient groups did not differ in personality and premorbid traits (Table 3).

**Table 3.** Personality, temperament, and premorbid traits of patients with short, medium, and long duration of anorexia nervosa (AN).

	Total sample inpatients with AN *n* = 169					
	<3 years	≥3 and <7 years	≥7 years	Test statistics		
	Short duration (SD-AN) *n* = 76	Medium duration (MD-AN) *n* = 47	Long duration (LD-AN) *n* = 46			
	Mean (SD)	Mean (SD)	Mean (SD)	*F*	*p*	Sheffè post hoc
TEMPS-A						
Depressive	12.2 (4.1)	12.7 (4.1)	13.2 (3.8)	0.74	0.480	–
Cyclothymic	9.9 (4.8)	9.6 (4.7)	9.9 (5.1)	0.01	0.916	–
Hyperthymic	6.9 (3.8)	6.1 (3.8)	7.8 (4.7)	1.7	0.187	–
Irritable	6.7 (3.9)	7 (3.6)	7.2 (4.3)	0.20	0.816	–
Anxious	12.8 (5.4)	13.8 (5.7)	13.8 (5.1)	0.55	0.577	–
FMPS	111.4 (25.8)	106.6 (27.3)	110 (26.3)	0.42	0.658	–
CHIRP						
Global perfectionism	2.8 (2.2)	2.8 (2.2)	3 (1.9)	0.08	0.919	–
Inflexibility	3.2 (1.4)	3.2 (1.4)	3.1 (1.1)	0.13	0.877	–
Need for order and symmetry	1.3 (1.5)	1.2 (1.3)	1.5 (1.5)	0.38	0.684	–
PCT-Q						
Harm avoidance	4.3 (2.9)	4.1 (2.7)	3.8 (3.2)	0.42	0.657	–
Alexithymia	2.5 (2.3)	2.9 (2.6)	2.3 (2.3)	0.78	0.460	–
Interoceptive awareness	2.7 (2.5)	2.6 (2.4)	2.5 (2.7)	0.14	0.866	–
Social phobia	1.8 (1.8)	2.3 (1.9)	1.9 (1.9)	1.11	0.330	–
Food obsessions	0.8 (1.2)	1 (1.3)	1.3 (1.6)	1.85	0.159	–

Abbreviations: CHIRP, Childhood Retrospective Perfectionism Questionnaire; FMPS, Frost Multidimensional Perfectionism Scale; PCT-Q, Premorbid Childhood Traits Questionnaire; TEMPS-A, Temperament Evaluation of Memphis, Pisa, Paris, and San Diego Autoquestionnaire.

### Clinical outcomes of hospitalization

The mean duration of hospitalization was 35.8 ± 17.1 days, with no differences across the groups (*F* = 2.03; *p* = 0.13), and all patients significantly improved after hospitalization on all measures except the EDI-2 body dissatisfaction subscale (Table 4).

**Table 4.** Outcome of hospitalization of patients with short, medium, and long duration of anorexia nervosa (AN).

	Total sample inpatients with AN *n* = 169														
	<3 years		≥3 and <7 years		≥7 years		Test statistics								
	Short duration (SD-AN) *n* = 76		Medium duration (MD-AN) *n* = 47		Long duration (LD-AN) *n* = 46		Main effect of group			Main effect of time			Time × group effect		
	T0	EOT	T0	EOT	T0	EOT	F	p	*η* ^2^	F	p	*η* ^2^	F	p	*η* ^2^
BMI	14.4 (1.9)	15.1 (1.6)	14.4 (1.7)	15.1 (1.6)	14.3 (2.1)	15.1 (2.1)	0.008	0.992	<0.001	124.53	**<0.001**	0.436	0.12	0.888	0.001
EDE-Q total score	3.4 (1.6)	2.9 (1.6)	3.4 (1.6)	2.3 (1.5)	3.2 (1.8)	2.5 (1.7)	0.47	0.623	0.008	58.82	**<0.001**	0.327	2.8	0.065	0.044
EDI-2															
DT	13 (7.8)	13.2 (7.6)	11.6 (8)	9.2 (7.8)	11 (8)	10.4 (8.2)	1.6	2.1	0.027	5.1	**0.026**	0.043	3.5	**0.034**	0.059
B	2.2 (3.5)	1.4 (2.6)	3.7 (5)	1.8 (3.7)	4.7 (6.3)	2.3 (3.2)	2.2	0.113	0.038	28.42	**<0.001**	0.202	2.7	0.073	0.046
BD	14 (7.5)	14.6 (7.6)	13.5 (7)	12.8 (6.4)	14.4 (6.8)	12.6 (7.1)	0.319	0.728	0.006	2.43	0.122	0.021	3.48	**0.034**	0.059
BDI	15.9 (8.5)	11.5 (7.7)	15.8 (7.3)	8.8 (6.8)	14.5 (8.2)	11.5 (9.1)	0.38	0.685	0.006	55.5	**<0.001**	0.322	2.9	0.60	0.047
STAI-state	54.9 (15.2)	52.4 (15.5)	53.7 (14.2)	47.2 (15.4)	55.1 (13.5)	51.8 (14.4)	0.614	0.543	0.010	13.55	**<0.001**	0.101	1.3	0.284	0.021
BSQ	119 (46.7)	117.5 (47.2)	114.6 (42.3)	94.3 (41.1)	110.5 (52.9)	102.3 (45.6)	1.13	0.327	0.021	15.9	**<0.001**	0.129	4.9	**0.009**	0.084
EQ-5D															
INDEX	0.42 (0.53)	0.65 (0.25)	0.54 (0.41)	0.69 (0.34)	0.43 (0.66)	0.6 (0.28)	0.66	0.517	0.012	14.88	**<0.001**	0.121	0.33	0.717	0.006
VAS	53.5 (26)	67.2 (19.8)	51.7 (17.2)	55.4 (24.8)	45.7 (23.3)	57.3 (21.8)	2.2	0.119	0.043	14.97	**<0.001**	0.134	1.5	0.230	0.030
HAM-A	19.9 (7.3)	15.4 (6.9)	17.9 (6.7)	14.2 (6.1)	19 (6.1)	12.7 (6.1)	1.4	0.248	0.019	102.47	**<0.001**	0.414	2.2	0.116	0.029
HAM-D	20.3 (7.5)	14.4 (6.8)	18.9 (7.1)	13.8 (6.9)	17.9 (7)	13.6 (6.6)	1.9	0.156	0.025	143.83	**<0.001**	0.498	0.4	0.673	0.005

Abbreviations: B, bulimia; BD, body dissatisfaction; BDI, Beck Depression Inventory; BMI, Body Mass Index; BSQ, Body Shape Questionnaire; DT, drive for thinness; EDE-Q, Eating Disorders Examination-Questionnaire; EDI-2, Eating Disorder Inventory-2; EQ-5D/VAS, EuroQoL Health Questionnaire/Visual Analogue Scale; HAM-A, Hamilton Rating Scale for Anxiety; HAM-D, Hamilton Depression Rating Scale; STAI, State Trait Anxiety Inventory.

**Figure 1.**
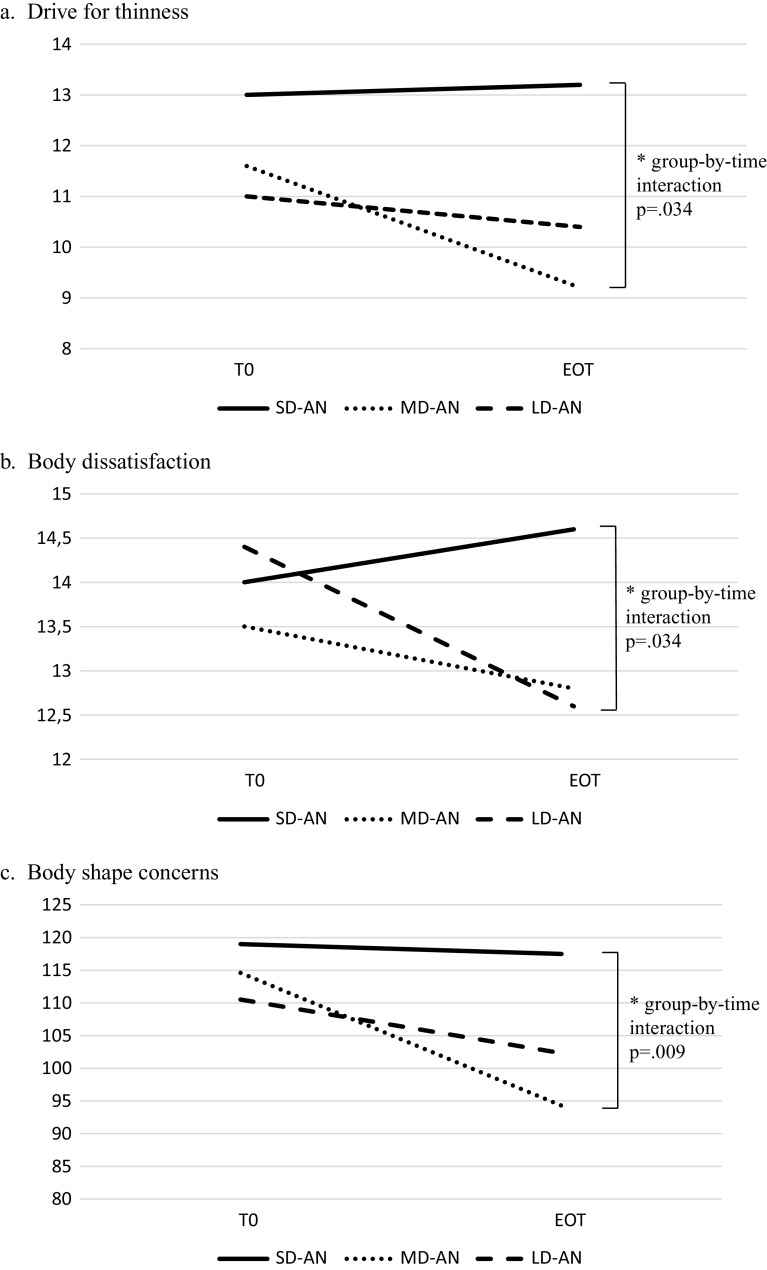
Trajectory of change during ehospitalization between admission (T0) and end of treatment (EOT) for patients with short (SD-AN: <3 years), medium (MD-AN: 3–6.99 years), and long (LD-AN: ≥7 years) duration of anorexia nervosa with respect to: (A) drive for thinness (as measured by the Eating Disorders Inventory-2); (B) body dissatisfaction (as measured by the Eating Disorders Inventory-2); and (C) body shape concerns (as measured by the Body Shape Questionnaire).

The three groups did not differ at baseline but showed different trajectories for drive for thinness and body dissatisfaction subscales of the EDI-2 and for body shape concerns on the BSQ. Those with SD improved poorly on the aforementioned parameters (see Figure 1). In contrast, no differences emerged across groups during hospitalization with respect to BMI, EDE-Q total score, EDI-2 bulimia subscale, depressive and anxiety symptoms (self-reported or interview-based), and QOL (Table 4).

Also, all three groups did not differ in therapeutic alliance at EOT (WAI-SR total SD, 67.4 ± 13.5; MD, 67.9 ± 12.9; LD, 70.8 ± 11.4; *F* = 0.76; *p* = 0.47).

When discharged, the groups did not differ in their stepping-down plans (*p* = 0.395; see Supplementary Material). Only three patients required a switch from voluntary to involuntary care.

## Discussion

Given the cogent need for a common definition of SE-AN to optimize treatment, we thoroughly compared patients with different AN durations to ascertain “profiles” of illness and hospitalization outcomes. After grouping patients according to their DOI, two main findings emerged. First, few differences were found across groups. Those with ≥7 years of AN were older, diagnosed mostly with AN-BP, and failed a higher number of AN-related treatments. Furthermore, those with a longer DOI reported a lower lifetime BMI and a trend toward a younger age at onset. Second, all patients responded equally well to the hospitalization; notably, patients with <3 years of AN improved less in drive for thinness and body-related concerns. Different from our a priori hypothesis, these novel and somehow unexpected findings show that DOI is an inadequate specifier for AN.

Patients’ current BMI was comparable across groups with different DOI, in line with previous research on outpatients [8,9], questioning a correlation between enduringness and severity of symptoms. Those with longer DOI (MD and LD) were diagnosed more frequently with BP-AN. However, patients’ phenotype cannot be an early severity index, since literature shows that many of those currently diagnosed with BP-AN tend to report a diagnostic cross-over from R-AN to BP-AN [37]. Also, patients with LD reported a trend toward a younger onset of AN; however, this finding did not survive post hoc correction so, although interesting, further studies are needed to confirm this result. In contrast with current BMI, participants’ lifetime BMI was lowest for those with LD when compared to those with SD. Therefore, despite current BMI, clinical and research attention should be paid to patients reaching a very low weight; in fact, duration and degree of severity of extreme emaciation during the course of AN should be investigated more. However, lowest lifetime BMI could be a time-dependent variable. In fact, as it is well-known that low weight is an unfavorable prognostic factor [38] our data highlight the relevance of working hard in treatment to avoid extremely low weights, as suggested by other data on evidence-based treatments for eating disorders [7].

Our data showed that all groups failed earlier interventions and, in keeping with other research showing patients with longer DOI as being frequent utilizers of the health care system [39] those with LD reported a higher number of previous hospitalizations. Furthermore, both MD and LD groups had greater therapeutic attempts using both medications and psychotherapy. Therefore, this finding adds to earlier literature a more detailed characterization of the failure of previous treatments. In fact, our data show not only that patients sought treatments on multiple occasions but also that they were offered (and then failed) these recommended treatments [35]. However, it should be borne in mind that the differences in health care systems across countries (i.e., availability of specialized centers, economic or insurance barriers) may affect these issues.

Interestingly, no differences in the severity of eating psychopathology emerged across groups, except for the bulimia subscale scores on the EDI-2, which were higher in those with a longer DOI who were also more frequently diagnosed with AN-BP. To this end, our findings are in line with earlier data on inpatients [7] while data on outpatients yields a contrasting result [9]. However, the same nonsignificant trend emerged also for anxiety and depressive symptomatology, in keeping with former studies [7,9]. This interesting finding fulfills a gap in the literature because we conducted a broad (i.e., clinician-based) assessment. Therefore, the current AN acute phase could level patients’ scores, and also a state-independent perspective could be adopted. In fact, depressive and anxious traits are putative vulnerability factors in AN [40,41] and may be independent of DOI. In line with earlier data [4] in our study those with LD showed a higher comorbidity profile; however, we confirmed this trend only for anxiety disorders. Given recent research on this topic [42], also comorbidity may be explored in the debate on specifiers in AN.

QOL is known to be severely impaired in inpatients with AN [43] and also has been proposed as potentially discriminating SE-AN [8,9]. Interestingly, our data do not seem to support the aforementioned findings, but a relevant caveat exists: Our sample was composed of patients in an acute phase of the disorder, so this characteristic may be similar for all patients independent of their clinical history. Moreover, different assessments may yield incomparable results.

We also designed this study to investigate stages of change and premorbid traits in SE-AN, which are important and emaciation-independent aspects. The former refers to the Stages of Change Model proposed by Prochaska and DiClemente [44] and assesses how prone patients with AN are to change various aspects of their symptomatology, entailing precontemplation, contemplation, preparation, action, or maintenance stages. The questionnaire on premorbid traits [33] investigated harm avoidance, social phobia, alexithymia, interoceptive awareness, and food obsessions as they appeared before the onset of AN. In line with the overall lack of differences across groups found in this study, the patients did not report differences in their AN stages of change, as also reported earlier [45]. Given the design of our study, we cannot rule out that patients fluidly shift into different stages of change according to their phase of AN; however, these data seem to indicate that acutely ill patients show similar stages of change.

In addition, as has been suggested previously [46], we focused on the analysis of premorbid characteristics but no temperament traits or premorbid conditions (including inflexibility and interoceptive awareness) differed across groups, suggesting the difficulty of early identification of individuals potentially at risk for developing SE-AN. This is even more true if we consider that groups did not differ in familiarity with psychiatric disorders, history of sexual abuse, and suicide attempts. Therefore, future research might shift focus from vulnerability factors to resilience skills.

Interestingly, our findings also suggest that patients with a longer DOI can respond equally well to acute treatments when anxiety, depression, and body-related concerns are included as outcomes. Follow-up data are needed to complete this picture; however, this finding is important because, in some countries, a label of SE-AN could hamper treatment delivery for patients [4] who could, according to our data, respond well (at least as much as could those with a shorter DOI) to intensive treatment. Furthermore, this data could illuminate how responsive those with a longer DOI can be to structured and short-term treatments, as other research has suggested [4,19]. In keeping with earlier research from our group [47] patients with LD showed a therapeutic alliance comparable to that of the younger ones. Interestingly, those with a shorter DOI improved less in the drive for thinness and body shape concerns, mirroring a network analysis showing patients with a shorter DOI reporting overvaluation of weight or shape as key elements [48].

Altogether, our data suggest that patients with a longer DOI were not “simply” more severe than were patients with a shorter history of AN. In agreement with studies advocating a broader investigation of patients with AN [9,16], we assessed a wide spectrum of dimensions, but still no differences emerged. In fact, DOI, psychometric assessments, and failure of previous treatments did not seem to sufficiently differentiate AN severity. Possibly, a clinimetric approach [49] may be required to make a shift from psychometric tests and “clinical wisdom” [50] to a more individualized and fine-grained evaluation. We hypothesize that clinicians may catch nuances that psychometric tests do not fully capture, eventually in line with other fields of medicine (e.g., Apgar scores are clinician based). Still, we raise the possibility that biologically informed measures may become available in the future era of precision psychiatry [51].

This study has some strengths, including the use of validated and widely applied self-report and interview-based assessments. However, some limitations exist. The sample was composed of inpatients in an acute phase of AN (thus eventually contributing to the lack of differences in symptom severity), and we applied a cross-sectional design. Taken together, findings from this study support the need for other “empirical tests to clarify the best-defining features of SE-AN” [5], eventually informed by the clinimetric approach. Notwithstanding, should these data be confirmed by larger studies, some clinical implications could be surmised, because we did not find “enduringness” to be a specifier of severity. Moreover, the effectiveness of hospitalization for patients with longer DOI was supported, while specific interventions on the core cognitive aspects of over-evaluation of body shape could be offered to patients with a shorter DOI.

## Data Availability

Data are not publicly available.

## Supplementary material

For supplementary material accompanying this paper visit http://dx.doi.org/10.1192/j.eurpsy.2021.2218. click here to view supplementary material
