# Supplementary material for: Inpatients with severe-enduring anorexia nervosa: Understanding the “enduringness” specifier
Source: Eur Psychiatry. 2021 Jun 21;64(1):e44. doi: 10.1192/j.eurpsy.2021.2218 (PMC8278247; doi:10.1192/j.eurpsy.2021.2218)
Supplement: Supplementary file 1 [file S0924933821022185sup001.docx]

|  | **Total sample**  **Inpatients with AN**  **n=169** | | | |
| --- | --- | --- | --- | --- |
|  | **< 3 years**  **Short duration**  **(SD-AN)**  **n=76** | **≥3 and < 7 years**  **Medium duration**  **(MD-AN)**  **n=47** | **≥ 7 years**  **Long duration**  **(LD-AN)**  **n=46** | **Test statistics** |
|  | N(%) | N(%) | N(%) | p |
| Discharge plan |  |  |  | .395 |
| Home | 36 (47.4) | 26 (55.3) | 22 (47.8) |  |
| Partial hospitalization | 16 (21.1) | 13 (27.7) | 9 (19.6) |  |
| Residential facility | 24 (31.5) | 8 (17) | 15 (32.6) |  |
